# Who’s for dinner? Bird prey diversity and choice in the great evening bat, *Ia io*

**DOI:** 10.1002/ece3.7667

**Published:** 2021-05-17

**Authors:** Lixin Gong, Biye Shi, Hui Wu, Jiang Feng, Tinglei Jiang

**Affiliations:** ^1^ Jilin Provincial Key Laboratory of Animal Resource Conservation and Utilization Northeast Normal University Changchun China; ^2^ Key Laboratory of Vegetation Ecology of Education Ministry Institute of Grassland Science Northeast Normal University Changchun China; ^3^ College of Life Science Jilin Agricultural University Changchun China

**Keywords:** bats, bird migration, foraging strategy, *Ia io*, molecular diet, predator–prey interaction

## Abstract

The mysterious predator–prey interaction between bats and nocturnally migrating birds is a very rare and incredible process in natural ecosystems. So far only three avivorous bat species, including two noctule bats (*Nyctalus lasiopterus* and *Nyctalus aviator*) and the great evening bat (*Ia io*), are known to regularly prey on songbirds during nocturnal avian migration. The information related to the diversity and the characteristics of the birds as prey and the hunting strategy in both species of noctule bats are already clear. However, the diversity of bird prey in the diet of *I. io* as confirmed by molecular identification remains unknown. Moreover, like hunting insects, it remains unclear whether the avivorous bats opportunistically prey on birds. Here, we used DNA metabarcoding to investigate the bird prey composition, diversity, and choice in diets of *I. io*. We found *I. io* consumed 22 species of seven families from Passeriformes with a body mass of 6–19 g, and preferentially selected small‐sized passerine birds for optimizing the benefit/risk trade‐off. Moreover, most of the species preyed upon were migratory birds, while four species were local resident birds, indicating that *I. io* may adopt both aerial‐hawking and gleaning strategies on songbirds as do the other two noctules. Further, *I. io* body mass did not influence in prey choice and predation richness on birds, suggesting *I. io* is an opportunistic avivorous predator. This study provides novel insights into the avian dietary ecology of *I. io* and completes the analysis of predator/prey interaction between three avivorous bats and nocturnally migrating birds. Our results also indicate bat predation on birds which occurs as an act of ecological opportunity may subject bats to intense natural selection pressure, causing them access to the new diet‐defined adaptive zones.

## Graphical abstract

We used DNA metabarcoding to investigate the bird prey composition, diversity, and choice in diets of the avivorous bat, *Ia io*. We found *I*. *io* consumed 22 species of seven families from Passeriformes. Moreover, *I*. *io* may adopt both aerial‐hawking and gleaning strategies on songbirds as do the other two noctules. *Ia io* body mass did not influence in prey choice and predation richness on birds, suggesting *I*. *io* is an opportunistic avivorous predator. Our work provides novel insights into the avian dietary ecology of *I*. *io* and completes the analysis of predator/prey interaction between three avivorous bats and nocturnally migrating birds.

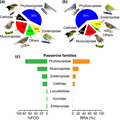

## 1 INTRODUCTION

Bats occupy unique night‐sky ecological niches, with more than 1,400 species nearly worldwide (Wilson & Mittermeier, 2019). The vast majority of animalivorous species mainly feed on arthropods, although some species also supplement their diets with small vertebrates, including fish, frogs, lizards, birds, mice, and smaller bats (Norberg & Fenton, 1988; Norberg & Rayner, 1987). Predation of bats on birds (namely, avivorous bats) is a rare process in nature and represents a case of dietary niche expansion from insects to birds, it has so far been found in 13 species belonging to 6 families (see Table S1). Most avivorous bats are mainly large bats, which occasionally capture resting birds using a gleaning foraging strategy (e.g., Vehrencamp et al., 1977). Only three temperate‐subtropical species are known to regularly prey on migrating birds but also prey on insects using an aerial‐hawking strategy. These include the greater noctule bat (*Nyctalus lasiopterus*) in Italy, Spain, and Russia (Dondini & Vergari, 2000; Ibáñez et al., 2001, 2016; Smirnov & Vekhnik, 2013), the birdlike noctule bat (*Nyctalus aviator*) in Japan (Fukui et al., 2013; Ibáñez et al., 2020), and the great evening bat (*Ia io*) in India and China (Han et al., 2007; Thabah et al., 2007).

So far, bats are the only mammals that have been reported to prey on nocturnally migrating passerines (Popa‐Lisseanu et al., 2007). The predation of bats on birds has probably influenced the evolution of bird migration strategies and antipredator behavior (Ibáñez et al., 2016). Thus, acquiring integral knowledge related to birds as prey to our understanding of predator–prey interaction between bats and migrating birds is critical. Traditional morphological analyses in droppings showed that *N. lasiopterus* and *I. io* captured only several species of birds in addition to insects (Dondini & Vergari, 2000; Han et al., 2007; Ibáñez et al., 2001; Thabah et al., 2007). For example, the diet of *N. lasiopterus* contained three small passerine birds: the European robin (*Erithacus rubecula*), the blue tit (*Cyanistes caeruleus*), and the wood warbler (*Phylloscopus sibilatrix*) (Dondini & Vergari, 2000; Ibáñez et al., 2001); meanwhile, one bird species, the Tickell's leaf warbler (*Phylloscopus affinis*) was presumably eaten by *I. io* (Han et al., 2007; Thabah et al., 2007). However, 31 bird species of eight families and 14 bird species of seven families from Passeriformes were identified in diets of *N*. *lasiopterus* and *N. aviator* by using molecular identification, respectively (Ibáñez et al., 2016, 2020). Thus, *N. lasiopterus* and *N. aviator* have been confirmed to prey mainly on nocturnal migrating birds on the wing and also only occasionally on resting birds in tree hollows or nest boxes (Dondini & Vergari, 2000; Fukui et al., 2013; Ibáñez et al., 2001, 2016, 2020; Smirnov & Vekhnik, 2013). However, the diversity of bird prey in the diet of *I. io* confirmed by molecular identification remains unknown.

More notably, molecular dietary analysis has shown that both *N*. *lasiopterus* and *N. aviator* prey seasonally on songbirds with a body mass of 5–25 g during their nocturnal migration (Ibáñez et al., 2016, 2020), and *N*. *lasiopterus* prefers to catch medium‐sized birds (10–15 g), with bird prey to bat body mass ratio averaging 25% (Ibáñez et al., 2016). The new prey to bat size threshold widely exceeded the traditional 5% threshold for bats hunting airborne prey (Fenton, 1990), which may explain why scientists were surprised and contested the emerging reports of predation strategy in bats (Andreas, 2010; Bontadina & Arlettaz, 2003). So far, only one study based on stable isotopes analyses has confirmed that *N. lasiopterus* preyed on a multitude of flying passerine birds during their nocturnal migratory journeys using an aerial‐hawking strategy (Popa‐Lisseanu et al., 2007). Thus, in order to amply confirm that bats employ an aerial‐hawking strategy to prey on birds, it is necessary to determine diversity and migratory patterns of bird prey in the diet of the all avivorous bats especially in *I. io*. Moreover, it is also important to clarify whether phenotypic attributes, such as body mass, influence prey choice and predation richness (PR) on birds in bats.

**FIGURE 1:**
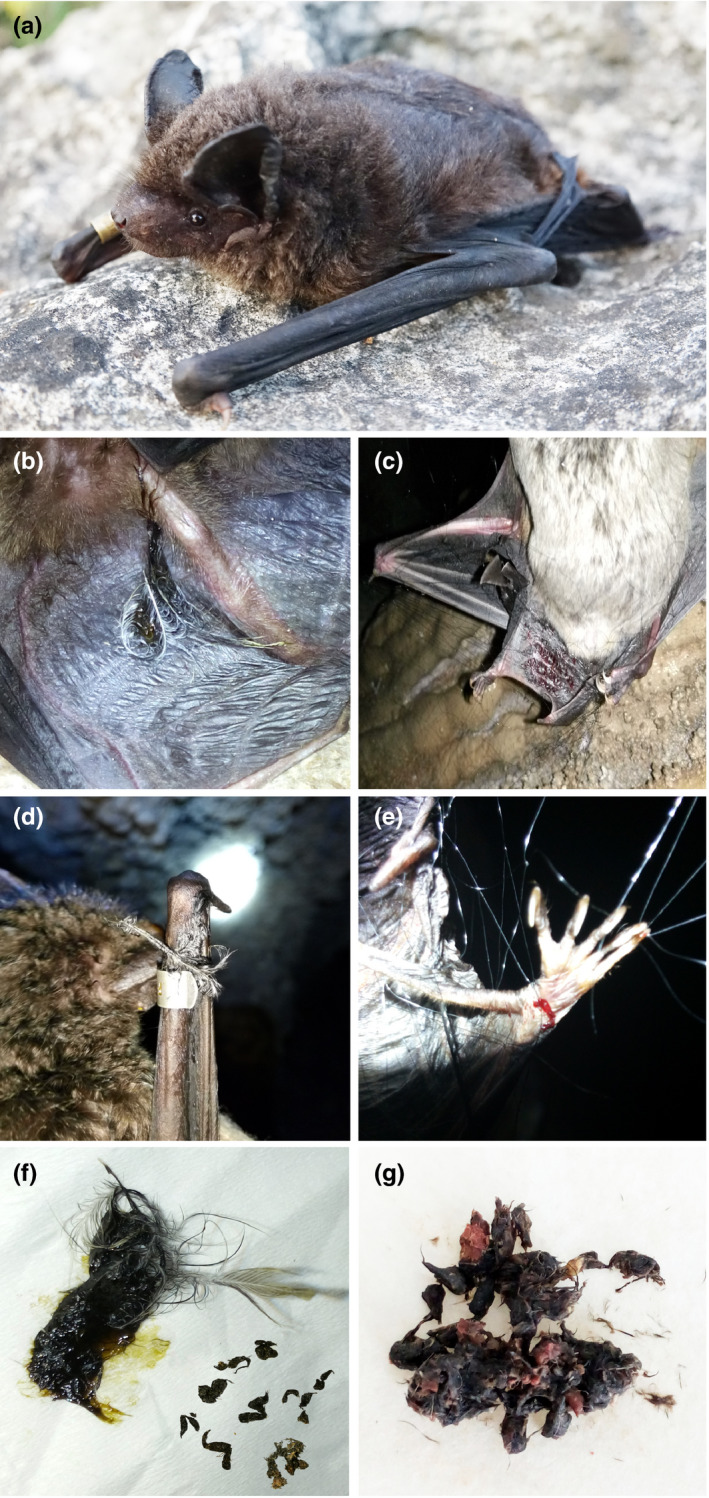
(a) A great evening bat, *Ia io* (Chiroptera: Vespertilionidae) captured from Xingyi City, Guizhou Province, China. (b–g) Evidence of *Ia io* predation on birds: (b) tail membrane carrying bird feathers; (c) residual blood on tail membrane; (d) bird feather clamped in the forearm with a mark ring; (e) bloodstain on a hind foot; (f) fecal pellets containing numerous feathers; (g) undigested muscle and bone fragments. Photos taken by Lixin Gong

*Ia io* (Figure 1a) is one of the largest and rarest species in the family Vespertilionidae and is widely distributed in Southeast Asia, northeastern India, and southern China. This species also is currently the only known bat to catch birds on the wing in southern China, where it mainly feeds on both insects and a passerine (*P. affinis*) based on traditional morphological identification (Han et al., 2007; Thabah et al., 2007). The present study employed DNA metabarcoding to determine the composition and diversity of bird prey species in *I. io*. Further, we investigated migratory patterns of bird prey, prey choice, and phenotypic constraints involved in bats preying on birds. Specifically, we tested the following hypothesis: (1) we hypothesized that a higher and/or different diversity of birds in the diet of *I. io* could be detected by using an advanced DNA metabarcoding technique when compared with the previous studies based on traditional morphological analyses; (2) because previous studies showed that *N*. *lasiopterus* and *N. aviator* prey on birds using mainly an aerial hunting strategy or occasionally using a gleaning strategy, we hypothesized that a large proportion of bird prey with migration habits in the diet of *I. io* would be observed; (3) since bats do show some selection of prey based on size and will usually avoid prey that are too large for them to capture/eat, and because *N. lasiopterus* only fed on birds with less than 25% of their own body mass (Ibáñez et al., 2016), we hypothesized that phenotypic attributes, such as body mass, would not influence prey choice and PR on birds in *I*. *io*, as long as birds being attacked fall below this threshold.

## 2 MATERIALS AND METHODS

### 2.1 Study site and sampling

The present study was conducted in Feilong Cave (24°58.426′N, 104°52.687′E) in Xingyi City, Guizhou Province, China. This is a mountainous region with elevations ranging from 1,500 to 2,200 m. This area has subtropical humid monsoon climate, with obvious characteristics of mountain climate. This typical karst cave is primarily surrounded by evergreen broad‐leaved forest, coniferous forest, shrub, grassland, and farmland. *Ia* *io* is the dominant bat species in this cave with a population size that decreased from about 2000 in 2007 to about 500 in 2011 (Han & He, 2012); currently, about 130 individuals still use the cave (L. Gong, personal observation) because of the effects of human activities and the exploitation of cave resources. In winter, *I. io* either go into hibernation in this cave or migrate farther south to overwinter elsewhere (personal observation by L. Gong). Several bat species, such as *Hipposideros armiger*, *Hipposideros pratti*, *Miniopterus fuliginosus*, and *Myotis chinensis* also roost seasonally in this cave.

Bats were captured using mist nets spread at cave entrances when the bats returned from foraging (between 20:00 and 07:00). Samples were collected at intervals of 1 to 2 days in three different entrances during a 12‐day period in every month to minimize interfering with the bats. Our sampling did not include pregnant individuals; no individuals were collected during the winter. The feather‐containing scats were collected from 43 individuals (40% of the total captured individual bats, *n* = 108) of *I. io* from March to November, 2017. Feather fragments were present in all fecal pellets of these 43 *I. io* and were estimated to form more than 90% of the fecal volume. The collections were divided into three seasons based on seasonal climatic periods in Guizhou (Zhang et al., 2014): spring (*n* = 9, March to early May), summer (*n* = 2, June to August), and autumn (*n* = 32, September to November). Each bat was placed individually in a clean and sterilized cotton bag for 30–60 min or until they defecated (less than two hours). Fecal pellets were collected from the bags and stored in 2‐ml cryo tubes filled with 100% ethanol. After bats emptied their feces, they were then sexed, weighed using an electronic balance (ProScale LC‐50, Accurate Technology, Inc., Asheville, NC, USA) to the nearest 0.01 g, and the forearm length of each individual was measured with a digital caliper (TESA‐CAL IP67, Tesa Technology, Renens, Switzerland) to the nearest 0.01 mm. In this case, body mass of bats was not affected by physiological condition (i.e., hungry status, pregnancy, hibernation). Finally, bats were tagged with a numbered split aluminum alloy bat ring (5.2 mm, Porzana Ltd., Icklesham, UK) on the right forearm for individual identification before release into the cave. Any recaptured individuals within the same season were excluded from fecal collection. Samples were short‐term refrigerated (0–4°C) until they were transported to the laboratory by dry ice, after which they were stored at –20°C until laboratory procedures.

### 2.2 DNA extraction, PCR amplification, and sequencing

All of the fecal pellets of individual bats were homogenized by using a grinder (TL2010S, DHS Life Science & Technology Co., Ltd., Beijing, China) prior to extraction. Next, the fecal mixtures were subsampled at 150–180 mg and the prey DNA was extracted using a QIAamp DNA Stool Mini Kit (Qiagen, Crawley, UK), according to the manufacturer's instructions with some modifications; the first heating suspension step at 70°C was increased from 5 to 20 min to aid in sufficient lysis, and final eluting was done in 60 μl of AE Buffer. Each extraction batch included an extraction blank to check for cross‐contamination. The content and quality of the extracted DNA product were determined using a NanoDrop 2000 UV‐vis spectrophotometer (Thermo Fisher Scientific, Wilmington, DE, USA). We preserved the extracted DNA at –20°C until PCR amplification.

For avian DNA analyses, a 380‐bp long fragment of the cytochrome c oxidase subunit I (COI) was amplified using a bird‐specific primer pair COIPreyFW (CGAGCAGARCTAGGCCAACC) and COIPreyRW (GCAGGCGGTTTTATGTTGATTGCTG) (Pastor‐Beviá et al., 2014). Both forward and reverse primers were tagged with an adapter, a pad, and linker sequencing. A unique barcode sequence was attached to the metabarcoding primers to permit the multiplexing of samples. Next, PCR amplification was performed using an ABI GeneAmp® 9,700 (ABI, Foster City, CA, USA) with all PCRs were performed following the protocol in Pastor‐Beviá et al. (2014). One‐two PCR blanks were included at each PCR series. Each sample was conducted in triplicate, and all were pooled together after the PCR amplification. All of the PCR products were visualized by electrophoresis using 2.0% agarose gel, then purified using an AxyPrep DNA Gel Extraction Kit (Axygen Scientific, Union City, CA, USA), and quantified with QuantiFluor^TM^‐ST (Promega, Madison, WI, USA) according to the manufacturer's protocol. Purified products were pooled in equimolar conditions and paired‐end sequenced (2 × 300) on an Illumina MiSeq platform (Illumina Inc., SanDiego, CA, USA) as described in the standard protocols by Shanghai Majorbio Bio‐Pharm Biotechnology Co., Ltd., China.

### 2.3 Sequence analysis and taxonomic identification

Raw sequences were quality‐filtered by Trimmomatic (Bolger et al., 2014) and merged by FLASH (Magoč & Salzberg, 2011). Valid sequences were obtained after quality control process, then dereplicated and excluded singletons sequences using Usearch (Edgar, 2010). The remaining sequences were clustered into MOTUs at 97% similarity thresholds using Usearch (Edgar, 2010), and chimeras also were removed simultaneously. We adopted a conservative approach for prey identification. The MOTUs with sequence numbers of <1% for avian samples of the total sequences in each sample were discarded in order to remove potentially erroneous and low‐abundance sequences. We used the reference database in BOLD (http://www.boldsystems.org/) and performed a BLAST in GenBank (https://www.ncbi.nlm.nih.gov/genbank/) for the taxonomic identification of representative sequences from each MOTU, basing on a > 98% of similarity threshold. Particularly, Hainan leaf warbler, *Phylloscopus hainanus*, matched 97.95% identity values and the high number of reads (12,834) only occupied one sample (if discarded, we would lose this sample). We thus regarded *Phylloscopus hainanus* as *Phylloscopus sp*. included in subsequent analysis. Those MOTUs not matching any reference sequence or not fulfilling the taxonomy were classified as unidentified.

### 2.4 Bird prey composition and diversity analyses

The bird prey composition in diets of *I*. *io* was quantified using percent of occurrence (POO) of bird species and prey items, as well as percent frequency of occurrence (%FOO) and relative read abundance (RRA) of prey families (Deagle et al., 2019). Avian taxonomic information was validated from *A Checklist on the Classification and Distribution of the Birds of China* (3rd ed.) (Zheng, 2017), and *HBW and BirdLife International Illustrated Checklist of the Birds of the World* *(Vol. 2): Passerines* (del Hoyo et al., 2016). We generated sample‐based bird prey species accumulation curves by calculating the interpolation and extrapolation curves of the Hill numbers *q* = 0 (species richness) using the iNEXT (Hsieh et al., 2016) package in R 3.4.4 (R Core Team, 2018). The 95% confidence interval was obtained by a bootstrap method based on 1,000 replications.

### 2.5 Bird prey migratory patterns analyses

Data classifying the status of prey birds as resident or migrant as well as the prey size of birds (estimated from the average body mass of pooled male and female individuals of each bird species) were obtained from *The Avifauna of Guizhou* (Wu et al., 1986) and *Fauna Sinica: Aves: Passeriformes* (Vols. 10, 12, 13, 14) (Zheng et al., 1995; 2010; 1982; 1998). To investigate the migratory patterns of bird prey species, we classified them as migratory birds (M, including S—summer visitor, W—winter visitor, and P—passing bird) and resident birds (R).

### 2.6 Prey choice and predation richness analyses

To probe bird prey size selection of *I. io*, we grouped the average body mass of each bird species into three categories (small size, <10 g; medium size, 10–15 g; and large size, >15 g) following Ibáñez et al. (2016). We then performed a simple linear regression to test the correlation between body mass of bats and birds. Here, we used body mass rather than forearm length instead as bat body size estimate, because bats will typically hunt their prey in flight based on the bat's body mass regardless of forearm length (Fenton, 1990). Additionally, our analysis found that no linear positive correlation existed between the forearm length of bats and body mass of birds (Figure S1). Data on migratory patterns and body mass were not obtained for two species identified at the genus level in the feces, *Phylloscopus sp*. and *Horornis sp*.; therefore, these were excluded from the above analyses.

We also calculated PR for 43 individual bats. The PR was defined as the number of different bird species that were preyed upon (i.e., the minimum number of birds) by each bat per night. The PR is considered that way for the sake of simplicity of the presented analysis, because it may not represent a single foraging night. For example, several factors may affect the digestion rate and DNA detectability such as temperature and even animal activity. To determine the key factors affecting PR, we conducted a generalized linear model (GLM) analysis with a Poisson distribution. Here, two individuals that consumed birds were excluded because most *I. io* individuals hunt insects in summer. In this model, we used the PR as a dependent variable, and body mass (BM) of bats and mean body mass of birds (MBMB) as independent variables. We used the corrected Akaike information criterion for small sample size (AICc) to obtain the optimized linear models using the dredge and model.avg functions in the MuMIn (Bartoń, 2017) package in R 3.4.4 (R Core Team, 2018).

## 3 RESULTS

### 3.1 On‐site evidence of predation on birds

**FIGURE 2:**
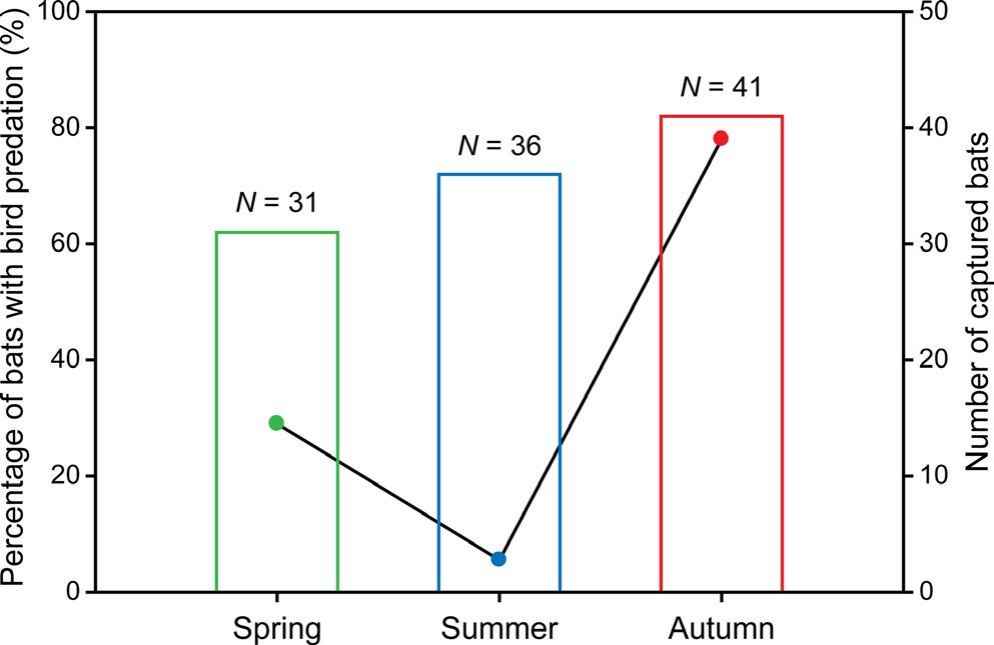
Percentage of individuals with bird predation evidence (Line and scatter plots) and number of captured individuals (histograms) of *Ia io* in each season

During the course of field sampling, we found some evidence of *I. io* predation on birds, including a tail membrane carrying bird feathers and residual blood, a bird feather caught in a mark ring, bloodstains on a bat's hind foot, fecal pellets containing numerous feathers, and undigested muscle and bone fragments found in the feces of some individuals (Figure 1b–g). Moreover, of the total captured 108 individual bats, these included 31 individuals from spring, 36 from summer, and 41 from autumn. And we found the percentage of bats with bird predation evidence per season 29% in spring, 6% in summer, and 78% in autumn (Figure 2).

### 3.2 Bird prey composition and species diversity in the diet of *I. io*

**FIGURE 3:**
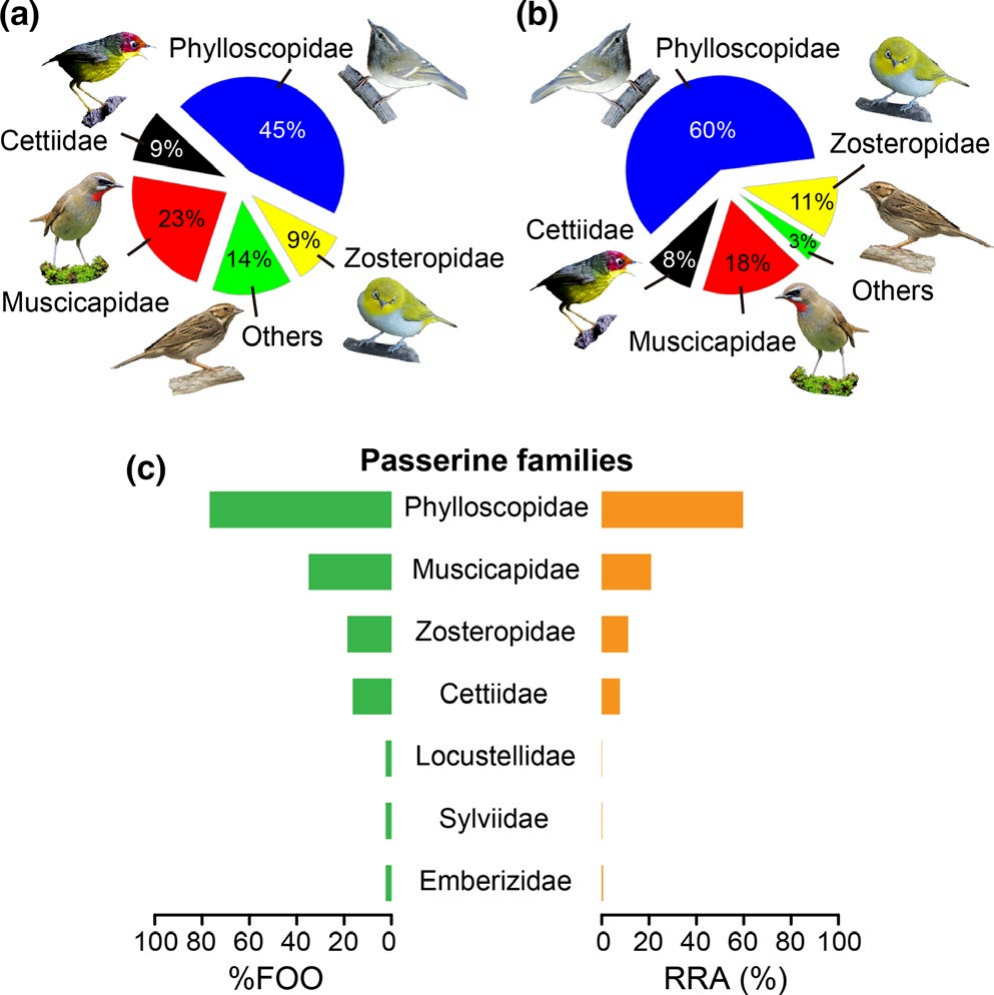
Bird prey composition and diversity in diet of *Ia io*. (a) Percent of occurrence (POO) representation of each bird family for bird species identified in *I. io* feces. (b) POO representation of each bird family for prey items identified in *I. io* feces. (c) Percent frequency of occurrence (%FOO) and relative read abundance (RRA) are expressed of passerine families in the diet of *I. io*. Bird images were licensed to download and cite from BIRDNET (https://www.birdnet.cn/; see the URL link and/or authors’ online moniker and personal homepage in Table S2)

Using bird‐specific primers, we identified a total of 85 bird prey items consumed by *I. io* belonged to 22 species of seven families of Passeriformes (Phylloscopidae, Muscicapidae, Cettiidae, Zosteropidae, Locustellidae, Sylviidae, and Emberizidae, listed based on the number of species found in each family from high to low; Table 1). The sample‐based species accumulation curve indicated that the 22 prey bird species represented most of the total diet, and a total richness at *c*. 26 prey species was estimated when extrapolating reached saturation (Figure S2). We found that the family Phylloscopidae dominated the diet of bats (60% of all prey items); therefore, this family represents the highest species diversity in the diet of bats (ten species). At the same time, only one species, *Phylloscopus inornatus* (Phylloscopidae), represented 23.5% of total prey items. The family Muscicapidae represented almost 18% of all prey items with a relatively high diversity in the diet (five species). The families Cettiidae and Zosteropidae together accounted for 19% of the total prey items with each represented by two species, respectively. The three remaining families added up to only 3% of all prey items; these contributed with only one bird prey species and one prey item (Figure 3a, b and Table 1). The occurrence summary (POO and %FOO) and read abundance summary (RRA) showed a similar pattern in the bird prey diet at the family‐level. The diet of *I. io* followed a descending order from the family Phylloscopidae (POO: 60%, %FOO: 77%, RRA: 60%), Muscicapidae (POO: 18%, %FOO: 35%, RRA: 21%), to the Zosteropidae (POO: 11%, %FOO: 19%, RRA: 11%) and other families (Figure 3b, c).

**TABLE 1:** Bird prey species identified in the diet of *Ia io*. Frequency: number of samples in which the species was identified (number of prey items)

Family	Genus and species	Similarity %	Frequency	Migration pattern	Body mass (g)
Phylloscopidae	*Phylloscopus coronatus*	99.72	3	P	8.70
	*Phylloscopus proregulus*	99.49	3	W	6.30
	*Phylloscopus armandii*	100	6	P, W	8.58
	*Phylloscopus ricketti*	99.49	2	P	7.00
	*Phylloscopus inornatus*	99.49	20	W	7.55
	*Phylloscopus trochiloides*	99.72	8	P	8.46
	*Phylloscopus fuscatus*	99.74	2	P	8.70
	*Phylloscopus subaffinis*	99.23	2	R	6.98
	*Phylloscopus hainanus/* *sp*.	97.95	2	—	—
	*Seicercus valentine*	99.49	3	R	8.55
Muscicapidae	*Ficedula albicilla*	99.74	2	P	10.90
	*Ficedula tricolor*	99.49	1	S	8.85
	*Larvivora cyane*	99.49	1	P	15.00
	*Calliope calliope*	99.23	8	P	18.50
	*Eumyias thalassinus*	99.23	3	S	17.80
Cettiidae	*Cettia castaneocoronata*	98.98	6	R	8.10
	*Horornis* *sp*.	98.97	1	—	—
Zosteropidae	*Zosterops japonicas*	99.24	6	S	10.75
	*Zosterops erythropleurus*	99.72	3	P, W	10.70
Locustellidae	*Locustella tacsanowskia*	99.22	1	S	12.75
Sylviidae	*Sinosuthora webbiana*	98.97	1	R	11.05
Emberizidae	*Emberiza pusilla*	99.74	1	W	15.30

Note

Migration patterns: type of resident or migrant, divided into migratory birds (M, including S—summer visitor, W—winter visitor, and P—passing bird) and resident birds (R). Body mass: estimated from the average body mass of pooled male and female individuals of each bird species.

### 3.3 Migratory patterns of bird prey

**FIGURE 4:**
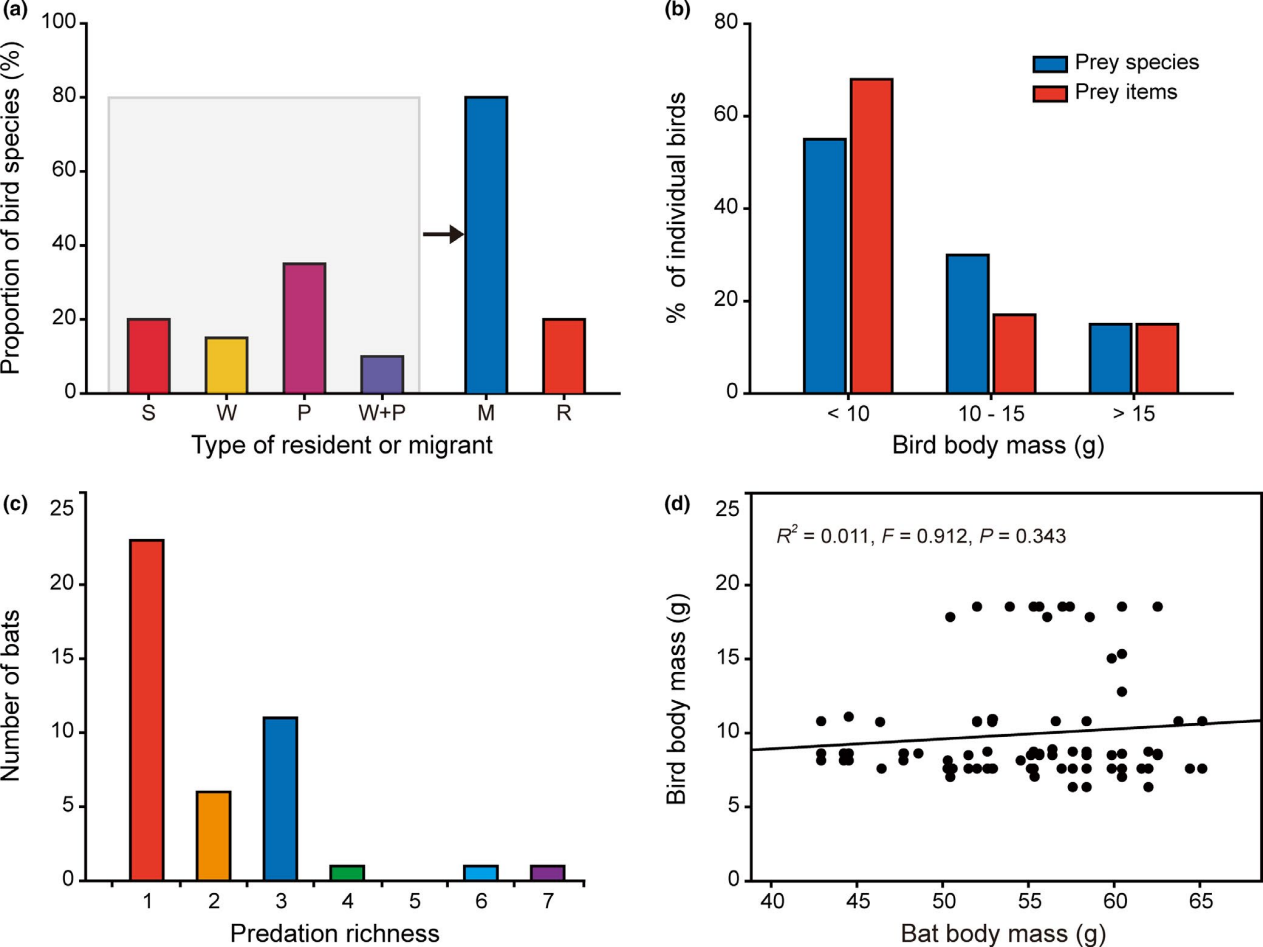
Migratory patterns of bird prey and *Ia io* prey choice. (a) Proportion of migratory patterns (type of resident or migrant) for bird species identified in feces of *I. io*. Migration patterns were divided into migratory birds (M, including S—summer visitor, W—winter visitor, and P—passing bird) and resident birds (R). (b) Frequency of bird body mass distribution for each category level of prey species and prey items identified in feces. (c) Number of different bird species detected in the feces of each 43 *I. io* individuals (predation richness). (d) Relationship between body mass of avivorous bats and body mass of birds

All of the identified bird prey species in the diet of *I. io* included songbirds found during migration (spring migration: March to early May; fall migration: September to November). Moreover, two species, *Phylloscopus armandii* and *Calliope calliope*, also were found during the breeding season (summer: June to August). The majority of prey species (80%, excluded two species without data; Figure 4a) and prey items (85%, excluded three prey items without data; Figure S3) were migratory birds (M, including S, W, P, W + P). Four species (20% prey species) were considered as local resident birds (R), which contributed only twelve prey items (15% prey items; Table 1, Figure 4a, and Figure S3).

### 3.4 Bird prey choice of *I. io*

The average body mass of the 20 prey species (excluded two species without data) was 10.53 ± 3.58 g, ranging from 6.3 g for *P. proregulus* to 18.5 g for *C. calliope*. The average body mass of the 82 identified prey items (excluded three prey items without data) was 9.97 ± 3.68 g (Table 1). In this case, an estimate of 6.3–18.5 g was considered as the bird body mass window of *I. io* (*n* = 43, mean body mass = 54.29 ± 5.84 g). Based on the choice of preference in body mass of 20 prey species and 82 prey items, we found that *I. io* tends to prey on small‐sized (<10 g) passerine birds (55% prey species and 68% of all prey items; Figure 4b). No significant linear positive correlation in body mass was found between bats and birds (Figure 4d), suggesting that bats prey on birds during opportunistic encounters when foraging under the precondition that they have ability to catch small birds.

### 3.5 Predation richness did not correlate with bat and bird prey body mass

The average PR on birds for each bird‐eating bat individual per night was 1.98 birds with a range from one to seven birds (Figure 4c and Figure S4). Model selection showed the null model was best supported (Table 2). Moreover, in the averaged model, BM of bats and MBMB also had no significant effects on PR (Table 3). These results indicate that PR of *I. io* was not related to the body mass of each bat and the body mass of birds which the bats preyed upon. This was consistent with the result of the relationship between body mass of bats and birds.

**TABLE 2:** Candidate linear models assessing the influence of body mass (BM) of bats and mean body mass of birds (MBMB) on predation richness (PR) of *Ia io*. The initial full model was of the form PR ~BM + MBMB

Model set	*df*	LogLik	AICc	Δ*_i_*	*w_i_*
Null	1	−65.29	132.68	0.00	0.45
BM	2	−64.60	133.53	0.85	0.30
MBMB	2	−65.27	134.86	2.18	0.15
BM, MBMB	3	−64.53	135.72	3.04	0.10

Note

Models are ranked by Akaike's information criterion corrected for small sample sizes (AICc) values, from the best to the worst model. In the model, PR was considered a dependent variable; BM and MBMB were independent variables. LogLik, Log likelihoods; Δ*_i_*, difference between the AICc of each model and the AICc of the best model; *w_i_*, Akaike weights.

**TABLE 3:** Model‐averaged parameter estimates of the best‐supported (before and including the null model) generalized linear models describing variation in predation richness with independent variables in *Ia io*

	Estimate	*SE*	Adjusted SE	*Z*	95% CI
(Intercept)	0.254	0.912	0.930	0.273	(−1.388, 2.044)
BM	0.023	0.019	0.020	1.142	(−0.016, 0.061)
MBMB	−0.010	0.037	0.038	0.261	(−0.081, 0.067)

Note

Parameters with 95% CI of all independent variables overlap with zero in all models.

Abbreviations: BM, bat body mass; MBMB, mean body mass of birds; SE, standard error; CI, confidence interval.

## 4 DISCUSSION

When compared with the results from previous studies (Han et al., 2007; Thabah et al., 2007), we found that the diet of *I. io* included higher bird prey diversity (at least 22 species), supporting our first hypothesis. Second, our results showed that the majority of bird prey species in the diet of *I. io* were migratory birds except for four species considered as resident birds, supporting our second hypothesis. These results suggested that when *I. io* prey on birds they mainly use an aerial hunting strategy or occasionally use a gleaning strategy. Finally, *I. io* body mass did not have an effect on prey choice and PR on birds, supporting our third hypothesis and suggesting that *I. io* is an opportunistic predator when capturing birds.

### 4.1 Evidence of *I. io* preying on birds

Do feathers in fecal pellets indicate that a bat has fed on birds? This question caused a scientific polemic and it has been proposed that additional evidence is needed to clarify the issue, such as knowing whether bone fragments would appear in a bat's feces (Andreas, 2010; Bontadina & Arlettaz, 2003; Ibáñez et al., 2003). Moreover, Bontadina and Arlettaz (2003) developed an accidental consumption hypothesis pointing out that the presence of feathers in feces could result from the accidental ingestion of free fluttering feathers in the air, or may occur because bats capture birds by concentrating on perches on marshes or wetlands during migration. So far, many studies have reported the discovery of feathers and bone fragments in bat feces (Dondini & Vergari, 2000; Fukui et al., 2013; Han et al., 2007; Ibáñez et al., 2001; Smirnov & Vekhnik, 2013; Thabah et al., 2007). Here, we also found undigested muscle in feces and direct evidence of the ingestion of birds, such as feathers and residual blood on the tail membrane of some bats. These findings strongly confirmed some *I. io* individuals had recently eaten the birds. Importantly, we found a mark ring on the forearm of a bat that was clamped on to a bird feather, inferring there may have been a fight between the bat and a bird and suggesting that birds displayed antipredator behavior in response to bats. Our findings further confirmed that bats can undoubtedly hunt birds rather than the occurrence of accidental consumption (Bontadina & Arlettaz, 2003) and presented empirical evidence supporting the hypothesis that nocturnally migrating bird species succumb to aerial‐hawking by several bat species (Dondini & Vergari, 2005; Ibáñez et al., 2016; Popa‐Lisseanu et al., 2007). However, further studies of the foraging ecology of these mysterious bats will be required to answer questions related to how they catch their bird prey and how this predatory behavior evolved (Dondini & Vergari, 2005).

### 4.2 Dietary bird composition and its potential benefits for fitness of *I. io*

Molecular analysis can complement previously used traditional morphological dietary data collection and provide deeper insights into the dietary ecology of wild animals. We found that *I. io* preys on a diverse variety of bird prey (at least 22 species) based on molecular dietary analysis, similar to findings for *N. lasiopterus* (Ibáñez et al., 2016) and *N. aviator* (Ibáñez et al., 2020), and *I. io* preferred to prey on species of the Phylloscopidae. However, here *P. affinis* was not found in the diet, indicating the morphological method of prey identification may not be accurate. This result was consistent with previous studies showing that *C. caeruleus* and *P. sibilatrix* also were not included in the diet of *N. lasiopterus* (Ibáñez et al., 2016). Additionally, these birds were not found in the diet of bats, possibly because of the low number of these birds near the studied colonies, resulting in a small chance of predation by bats. In particular, we clearly found that *I. io* prey on many species of Passeriformes to attain their optimal diet in spring and autumn. The size and/or nutrition in prey species of Passeriformes are actually higher than those of other prey species (i.e., insects) (Popa‐Lisseanu et al., 2007). The choice of food in *I. io* may be designed to maximize the intake of energy and protein before and after hibernation, which can be proposed as a food quality hypothesis. However, whether the food quality hypothesis is associated with the protein maximization theory (Mattson, 1980) and/or energy maximization theory (Schoener, 1971) of animal foraging strategy still needs to be further verified.

### 4.3 Migratory patterns of bird prey and its implication for hunting strategy

In addition to mainly preying on birds mostly during migration, *I. io* also preys on a small number of local resident birds, as well as two migratory bird species (*P. armandii and C. calliope*) during the breeding period. This finding was also consistent with previous studies of *N. lasiopterus* and *N. aviator* showing that their diet included both migratory and sedentary birds (Ibáñez et al., 2016, 2020). Many species are migratory, but do not necessarily migrate directly through the study area; these birds may also be preyed upon when they could be reproducing or wintering in the area. These sedentary birds may be preyed upon as a result of their nocturnal (dispersal) movements (Mukhin et al., 2009; Ward et al., 2014; Zheng, 1995). Some diurnal songbirds or resident birds will perform nocturnal activity patterns or nondirectional and short‐distance migration according to habitat, climate, and seasonal changes. For example, nocturnally migrating Eurasian reed warbler (*Acrocephalus scirpaceus)* use nocturnal movements at other times of year, including during the breeding season (Mukhin et al., 2009). Thus, *I. io* may behave like two other noctules that may hunt birds either mainly by using an aerial hunting strategy during the nocturnal migration of birds or occasionally by using a gleaning strategy while the birds are resting at night (Dondini & Vergari, 2000; Ibáñez et al., 2016, 2020). However, future behavioral studies should be carried out to clarify this issue.

### 4.4 Prey choice and an opportunistic avivorous predator

We found *I. io* preferentially selected smaller species of the family Phylloscopidae and small‐sized (< 10 g) passerine birds. This was inconsistent with *N. lasiopterus*, which selected medium‐sized bird species (10–15 g) (Ibáñez et al., 2016), thereby presumably to more optimize the benefit trade‐off between energy intake and predation risk. This may not be explained by differences in body size of bats because the average body mass of *I. io* (54.3 g) was slightly bigger than that of *N. lasiopterus* (52.0 g) (Ibáñez et al., 2020). Moreover, here the nonsignificant relationship between body mass of *I. io* and body mass of birds may also support the view. Thus, the disparity in the bird prey size choice between *I. io* and *N. lasiopterus* probably occurs because of the difference in species types and size of the nocturnal passerine migrants in different regions. *Ia io* has an average bird prey to bat body mass ratio of 18.4% (ranged from 11.6%–34.1%), conforming to a new threshold (<25%) (Ibáñez et al., 2016), but widely exceeded the 5% threshold in respect of bats hunting airborne prey (Fenton, 1990). Ibáñez et al. (2003) suggested that the 5% threshold rule remains valid for bats hunting for flying insects, but not for these bats that hunt for birds at high altitudes. The avivorous bats have a relatively large body size, low frequency echolocation calls, and high wing loading, as well as being adapted for rapid flight and for detecting and catching relatively large prey in open spaces (Fukui et al., 2004, 2011; Ibáñez et al., 2001; Thabah et al., 2007). Moreover, the large body size, strong skull, and high bite force (Shi et al., 2020) combined with a long‐distance echolocation system of *I. io*, could allow it to exploit a recently found feeding niche (nocturnally migrating songbirds). Birds may be easier to detect than insects for these bats at a greater spatial range. This supports the hypothesis stating that detecting small passerine birds would be similar to locating large moths from an acoustic perspective (Ibáñez et al., 2003). However, obviously only further behavioral experiments will help in answering how bats prey on birds by an aerial‐hawking strategy.

Optimal foraging theory predicts that prey size of a predator depends on the predator's own size (Stephens & Krebs, 1986). That is, in any species of a given species pool (terrestrial, aquatic, or marine), prey body size and feeding range increase with an increase in the body size of the predators (Gravel et al., 2013). However, our study found that no significant positive correlation exists in body mass between *I. io* and bird prey, suggesting that *I. io* is an opportunistic avivorous predator under the precondition that they have ability to catch small birds. This may explain why some individual bats could catch more than two and up to seven bird species in one night, while the PR was also not affected by a bat's own body mass and captured bird body mass. However, for gleaning foragers, the bats could capture even more prey and actually eat them on the substrate or ground because they experience less risk of predation and injury. For example, the frog‐eating bat (*Trachops cirrhosis*) consumed as many as six small frogs per hour (Ryan et al., 1981). Thus, to be a generalist may be beneficial for avivorous bats because of the nature of high energy cost for hunting migrating birds. The future studies should determine that whether a predation threshold of body size exists for bats hunting for birds in flight.

## CONFLICT OF INTEREST

The authors declare no conflict of interest.

## AUTHOR CONTRIBUTIONS

**Lixin Gong:** Conceptualization (equal); Data curation (equal); Formal analysis (lead); Investigation (equal); Methodology (lead); Visualization (lead); Writing‐original draft (lead). **Biye Shi:** Conceptualization (equal). **Hui Wu:** Funding acquisition (equal); Writing‐review & editing (supporting). **Jiang Feng:** Funding acquisition (supporting); Resources (lead). **Tinglei Jiang:** Conceptualization (equal); Data curation (equal); Funding acquisition (equal); Project administration (lead); Supervision (lead); Writing‐review & editing (lead).

## ETHICAL APPROVAL

Bat capture methods and experiments in this study conformed to the Northeast Normal University Guidelines for Animal Research. All experimental procedures adhered to the ASAB/ABS Guidelines for the Use of Animals in Research and were approved by the Wildlife Conservation Office of the Jilin Forestry Department, China.

## Supporting information

Appendix S1

## Data Availability

Data used in this study are available at Dryad Digital Repository (https://doi.org/10.5061/dryad.3tx95x6g5).
