# Supplementary material for: Who’s for dinner? Bird prey diversity and choice in the great evening bat, Ia io
Source: Ecol Evol. 2021 May 17;11(13):8400–9. doi: 10.1002/ece3.7667 (PMC8258197; doi:10.1002/ece3.7667)
Supplement: Supplementary file 1 — Appendix S1 [file ECE3-11-8400-s001.docx]

**Supplementary Information for**

**Who’s for dinner? Bird prey diversity and choice in the great evening bat, *Ia io***

**Table S1.** List of avivorous bats from publications records and their Foraging strategies.

| **Family** | **Species** | **Foraging strategy** | **Reference** |
| --- | --- | --- | --- |
| Pteropodidae | *Hypsignathus monstrosus* | Gleaning foraging strategy | van Deusen, 1968; a |
| Nycterididae | *Nycteris grandis* | Gleaning foraging strategy | Fenton, Thomas, & Sasseen, 1981; Fenton et al., 1993 |
| Megadermatidae | *Megaderma spasma* | Gleaning foraging strategy | Balete, 2010 |
|  | *Macroderma gigas* | Gleaning foraging strategy | Boles, 1999 |
| Phyllostomidae | *Vampyrum spectrum* | Gleaning foraging strategy | Vehrencamp, Stiles, & Bradbury, 1977 |
|  | *Chrotopterus auritus* | Gleaning foraging strategy | Sazima, 1978; Medellín, 1988 |
|  | *Phyllostomus hastatus* | Gleaning foraging strategy | Santos, Aguirre, Vázquez, & Ortega, 2003 |
|  | *Trachops cirrhosus* | Gleaning foraging strategy | Bonato, Facure, & Uieda, 2004 |
|  | *Tonatia bidens* | Gleaning foraging strategy | Martuscelli, 1995 |
| Hipposideridae | *Hipposideros diadema* | Gleaning foraging strategy | Pavey & Burwell, 1997 |
| Vespertilionidae | *Nyctalus lasiopterus* | Aerial-hawking strategy  Gleaning foraging strategy | Dondini & Vergari, 2000; Ibáñez, Juste, Garcia-Mudarra, & Agirre-Mendi, 2001 |
|  | *Nyctalus aviator* | Aerial-hawking strategy  Gleaning foraging strategy | Fukui, Dewa, Katsuta, & Sato, 2013; Ibáñez et al., 2020 |
|  | *Ia io* | Aerial-hawking strategy | Han, Gu, Liang, & Zhang, 2007;  Thabah et al., 2007 |

a. Anecdotal report

**Table S2.** Lists of URL links and/or authors’online moniker and personal homepage of bird images that were used in Figures 1a and b which were downloaded and cited from BIRDNET (https://www.birdnet.cn/).

| **Bird images** | **Species** | **URL link and/or author’s online moniker and** **personal homepage** |
| --- | --- | --- |
| 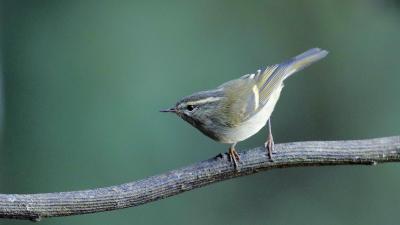 | *Phylloscopus inornatus* | Chengdu Laowu  <https://www.birdnet.cn/?46795> |
| 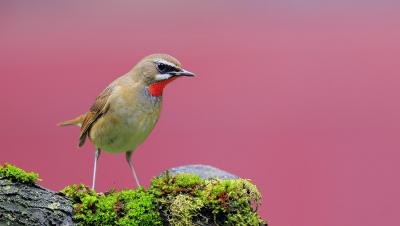 | *Calliope calliope* | Chengdu Laowu  <https://www.birdnet.cn/?46795> |
| 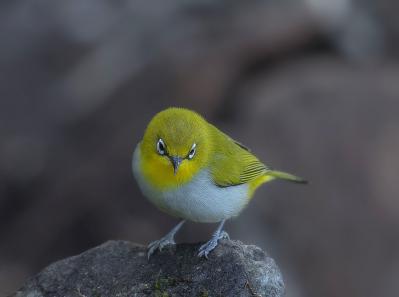 | *Zosterops japonicus* | <https://www.birdnet.cn/atlas.php?mod=show&action=atlasinfo&aid=75572>  Mingge1962  https://www.birdnet.cn/?91448 |
| 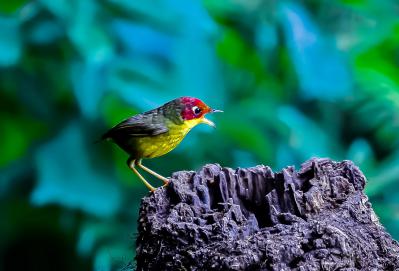 | *Cettia castaneocoronata* | Gushan  <https://www.birdnet.cn/?83487> |
| 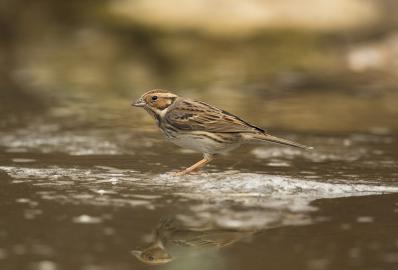 | *Emberiza pusilla* | https://www.birdnet.cn/atlas.php?mod=show&action=atlasinfo&aid=127061  Guangying Suixin  https://www.birdnet.cn/?183940 |

**
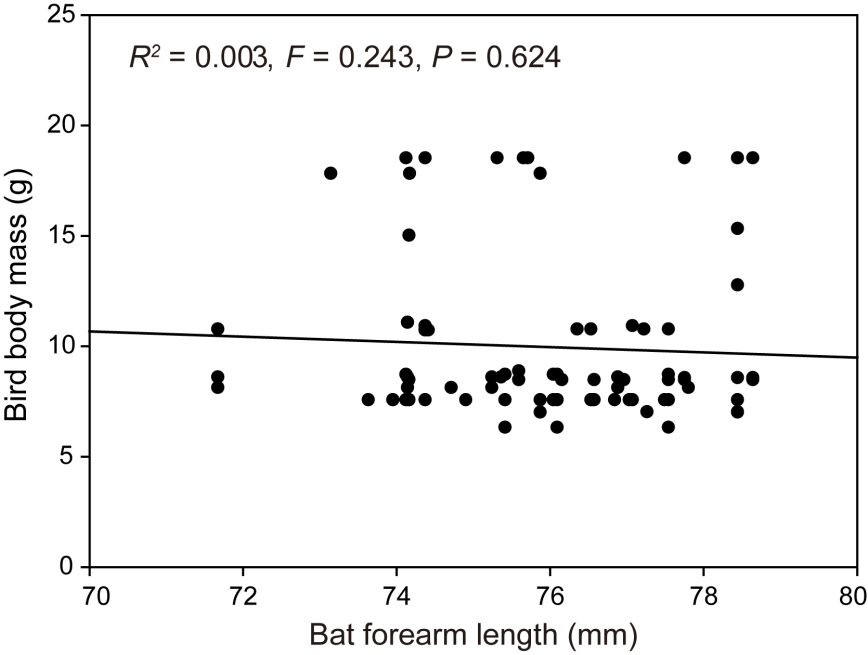
**

**Figure S1.** Relationship between forearm length of avivorous bats and body mass of birds.


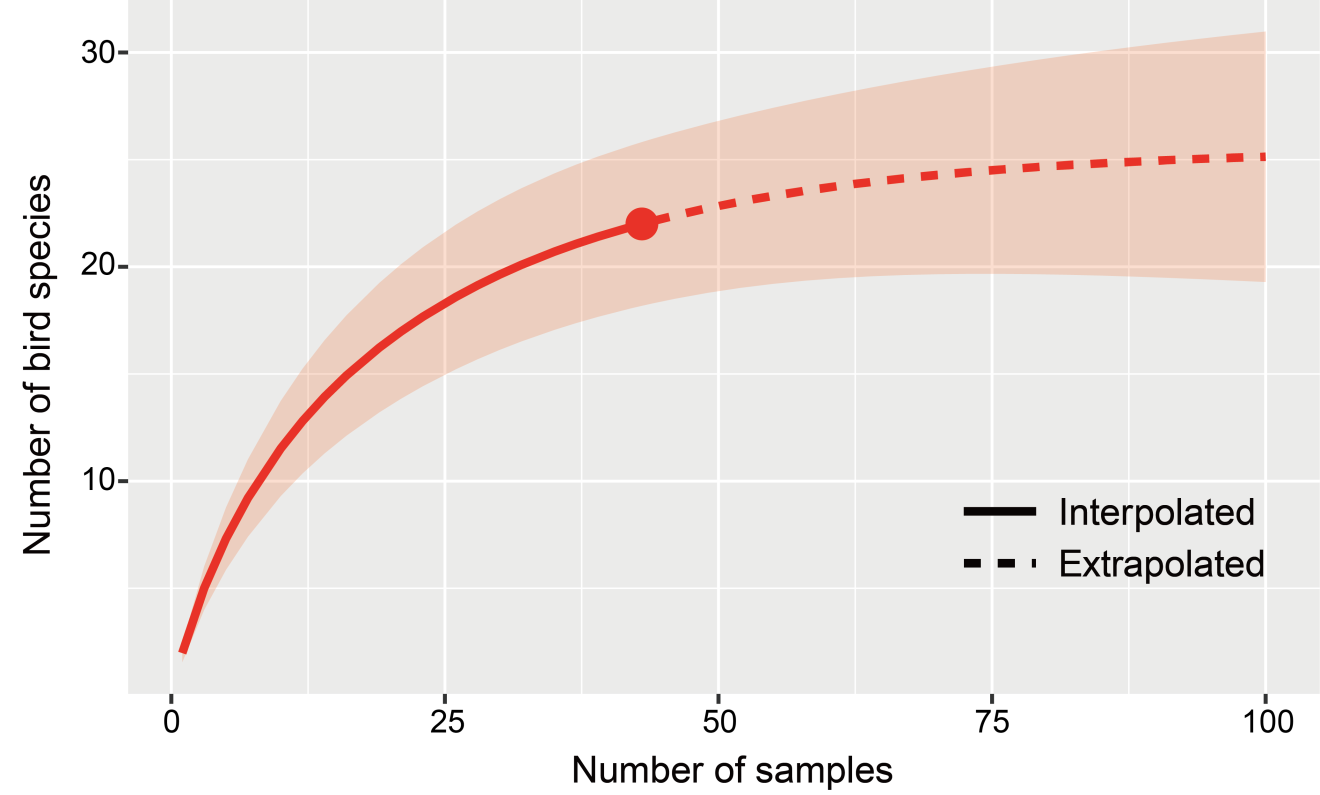


**Figure S2.** Sample-based interpolation and extrapolation curve with 95% confidence intervals (shaded areas) for estimated species richness of bird prey of *Ia io*. Curve was extrapolated to 100 of the base sample size. The solid circle represents the reference samples.


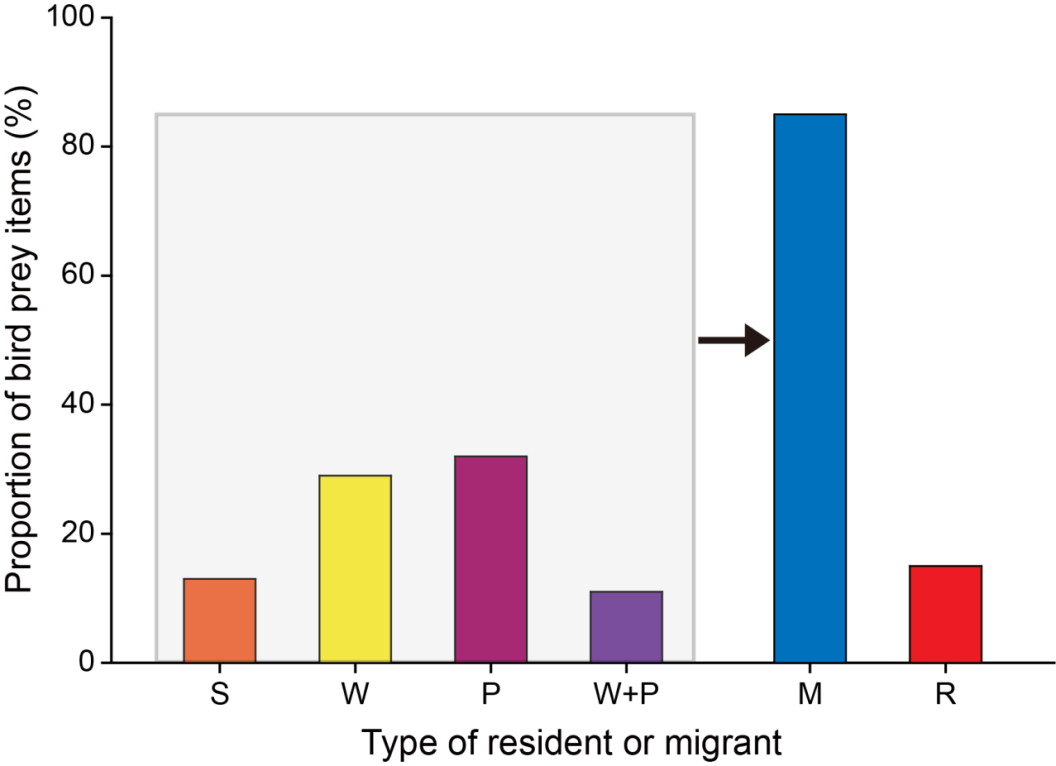


**Figure S3.** Proportion of migratory patterns (type of resident or migrant) for bird prey items identified in feces of *Ia io*. Migration patterns were divided into migratory birds (M, including S-summer visitor, W-winter visitor, and P-passing bird) and resident birds (R).


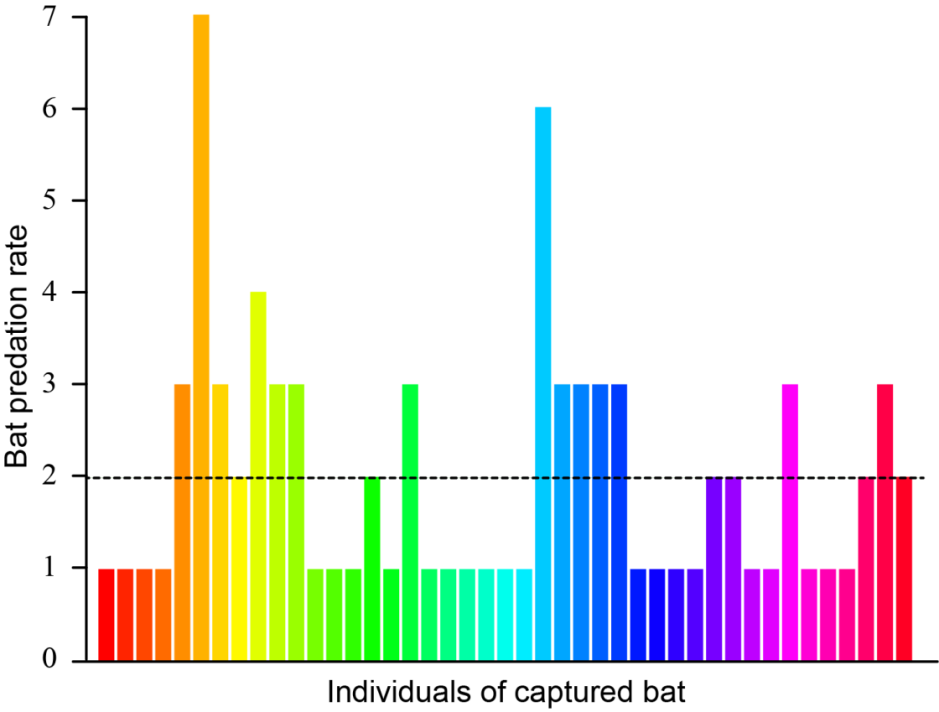


**Figure S4****.** Identifiable number of bird species in 43 individual *Ia io* bat feces. Namely predation intensity or rate of each bat per night. The dotted line represents the average predation rate (mean ± SE: 1.98 ± 0.21, range 1–7).

**Supplementary references**

Balete, D. S. (2010). Food and roosting habits of the lesser false vampire bat, *Megaderma spasma* (Chiroptera: Megadermatidae), in a Philippine lowland forest. *Asia Life Sciences, 4*(4), 111–129.

Boles, W. E. (1999). Avian prey of the Australian Ghost Bat *Macroderma gigas* (Microchiroptera: Megadermatidae): prey characteristics and damage from predation. *Australian Zoologist, 32*(1), 82–91.

Bonato, V., Facure, G. K., & Uieda, W. (2004). Food Habits of Bats of Subfamily Vampyrinae in Brazil. *Journal of Mammalogy, 85*(4), 708–713.

Dondini, G., & Vergari, S. (2000). Carnivory in the greater noctule bat (*Nyctalus lasiopterus*) in Italy. *Journal of Zoology, 251*(2), 233–236.

Fenton, M. B., Rautenbach, I. L., Chipese, D., Cumming, M. B., Musgrave, M. K., Taylor, J. S., & Volpers, T. (1993). Variation in foraging behaviour, habitat use, and diet of Large Slit-faced bats (*Nycteris grandis*). *Z. Säugetierkunde, 58*, 65–74.

Fenton, M. B., Thomas, D. W., & Sasseen, R. (1981). *Nycteris grandis* (Nycteridae): an African carnivorous bat. *Journal of Zoology, 194*(4), 461–465.

Fukui, D., Dewa, H., Katsuta, S., & Sato, A. (2013). Bird predation by the birdlike noctule in Japan. *Journal of Mammalogy, 94*(3), 657–661.

Han, B., Gu, X., Liang, B., & Zhang, S. (2007). Bird Predation and Selection of Different Insects by Great Evening Bats (*Ia io*). *Zoological Research, 28*(3), 243–248.

Ibáñez, C., Fukui, D., Popa-Lisseanu, A. G., Pastor-Beviá, D., García-Mudarra, J. L., & Juste, J. (2020). Molecular identification of bird species in the diet of the bird-like noctule bat in Japan. Journal of Zoology. doi:10.1111/jzo.12855

Ibáñez, C., Juste, J., Garcia-Mudarra, J. L., & Agirre-Mendi, P. T. (2001). Bat predation on nocturnally migrating birds. *Proceedings of the National Academy of Sciences of the United States of America, 98*(17), 9700–9702.

Martuscelli, P. (1995). Avian predation by the Round-eared Bat (*Tonatia bidens*, Phyllostomidae) in the Brazilian Atlantic forest. *Journal of Tropical Ecology, 11*, 461–464.

Medellín, R. A. (1988). Prey of *Chrotopterus auritus*, with Notes on Feeding Behavior. *Journal of Mammalogy, 69*(4), 841–844.

Pavey, C. R., & Burwell, C. J. (1997). The diet of the diadem leaf-nosed bat *Hipposideros diadema*: confirmation of a morphologically-based prediction of carnivory. *Journal of Zoology, 243*(2), 295–303.

Santos, M., Aguirre, L. F., Vázquez, L. B., & Ortega, J. (2003). Phyllostomus hastatus. *Mammalian Species, 722*, 1–6.

Sazima, I. (1978). Vertebrates as Food Items of the Woolly False Vampire, *Chrotopterus auritus Journal of Mammalogy, 59*(3), 617–618.

Thabah, A., Li, G., Wang, Y. N., Liang, B., Hu, K. L., Zhang, S. Y., & Jones, G. (2007). Diet, echolocation calls, and phylogenetic affinities of the great evening bat (*Ia io*; vespertilionidae): another carnivorous bat. *Journal of Mammalogy, 88*(3), 728–735.

van Deusen, H. M. (1968). Carnivorous Habits of *Hypsignathus monstrosu*. *Journal of Mammalogy, 49*(2), 335–336.

Vehrencamp, S. L., Stiles, F. G., & Bradbury, J. W. (1977). Observations on the Foraging Behavior and Avian Prey of the Neotropical Carnivorous Bat, *Vampyrum spectrum*. *Journal of Mammalogy, 58*(4), 469–478.
